# Blood-based transcriptomic biomarkers for response to [^177^Lu]Lu-DOTA-TATE therapy in neuroendocrine tumors

**DOI:** 10.1186/s13550-025-01284-w

**Published:** 2025-08-02

**Authors:** Hyunpil Sung, Sungwoo Bae, Minseok Suh, Hongyoon Choi, Hyung-Jun Im, Gi Jeong Cheon, Keon Wook Kang

**Affiliations:** 1 Department of Nuclear Medicine, Seoul National University Hospital, 03080 Seoul, Republic of Korea; 2 Department of Molecular Medicine and Biopharmaceutical Sciences, Graduate School of Convergence Science and Technology, Seoul National University, Seoul, Republic of Korea; 3 Radiation Research Institute, Seoul National University College of Medicine, Seoul, Republic of Korea; 4 Portrai, Inc., 78-18, Dongsulla-gil, Jongno-gu, 03136 Seoul, Republic of Korea; 5 Department of Nuclear Medicine, Seoul National University College of Medicine, 03080 Seoul, Republic of Korea; 6 Department of Biomedical Sciences, Seoul National University Graduate School, Seoul, Republic of Korea

**Keywords:** Neuroendocrine tumor, RNA sequencing, Blood biomarker, [^177^Lu]Lu-DOTA-TATE

## Abstract

**Background:**

[^177^Lu]Lu-DOTA-TATE is an effective treatment for metastatic neuroendocrine tumors (NETs) expressing somatostatin receptors. While the tumor uptake [^177^Lu]Lu-DOTA-TATE of has shown potential as a predictive biomarker, patient response to the treatment varies significantly. In this study, we aim to identify a predictive blood-based transcriptomic biomarker to better understand individual responses to [^177^Lu]Lu-DOTA-TATE therapy.

**Results:**

Twenty-six patients were prospectively enrolled in this study. Responders were defined as patients who showed partial response or stable disease and non-responders were defined as patients who showed progressive disease according to RECIST1.1 criteria. Of the 26 patients, responders (n = 20) exhibited distinct gene expression profiles compared to non-responders (n = 6). Among the 21 differentially expressed genes identified between the groups, 13 genes were upregulated in non-responders and were associated with the innate immune system. Weighted Gene Co-expression Network Analysis identified a significant gene module linked to treatment response, with eEF1A1 emerging as a key hub gene correlated with favorable outcomes. Baseline clinical and laboratory parameters did not differ significantly according to treatment response.

**Conclusions:**

This study identifies specific blood transcriptomic profiles associated with the innate immune response and a key hub gene linked to treatment outcomes, suggesting an immune-related component in response to [^177^Lu]Lu-DOTA-TATE therapy. These findings may guide patient selection based on systemic immune markers and inform future therapeutic strategies.

**Supplementary Information:**

The online version contains supplementary material available at 10.1186/s13550-025-01284-w.

## Background

Peptide receptor radionuclide therapy (PRRT) with [^177^Lu]Lu-DOTA-TATE is a favorable treatment option for patients with metastatic neuroendocrine tumors expressing high levels of somatostatin receptors (SSTR). [^177^Lu]Lu-DOTAT-ATE has been shown to extend progression-free survival with limited acute toxicity in patients with progressive midgut NETs [1]. Additionally, following the promising results of the NETTER-2 trial, which included [^177^Lu]Lu-DOTA-TATE as a first-line treatment for advanced grade 2 and 3 gastroenteropancreatic (GEP) NETs, it has become an important treatment option for metastatic NETs [2].

One of the key advantages of [^177^Lu]Lu-DOTATATE therapy is that eligibility can be assessed with pretreatment positron emission tomography (PET) scans using the Krenning score, which evaluates SSTR2 expression [3, 4]. This expression level, measured by PET, correlates with DOTA-TATE uptake in tumors and the absorbed dose, ultimately impacting therapeutic efficacy [5, 6]. Thus, predictive biomarkers based on pretreatment PET scans or dosimetry from post-treatment [^177^Lu]Lu-DOTA-TATE, offer a robust method for predicting response and enabling personalized treatment approaches [7]. However, due to the complex pathophysiology and tumor heterogeneity of NETs, along with variations in the tumor microenvironment, neither absorbed dose nor pretreatment PET alone are sufficient for predicting therapeutic outcomes [8]. Additionally, the use of dosimetry has primarily focused on determining the appropriate dose, with limited evidence linking it directly to patient outcomes [9]. Therfore, additional biological and systemic profiles are essential to enable a more precise, personalized approach to NET treatment with [^177^Lu]Lu-DOTA-TATE, especially considering the availability of other therapeutic options which target various tumor characteristics.

In this study, we aimed to identify systemic factors in blood markers associated with response to [^177^Lu]Lu-DOTA-TATE using RNA-sequencing. These discovered markers and hub genes could not only serve as predictive biomarkers to better predict treatment response, but also enhance the systemic effects of PRRT and potentially address PRRT resistance. To date, few studies have focused on predicting [^177^Lu]Lu-DOTA-TATE response across different patients. For example, the PRRT predictive quotient has shown high accuracy for predicting treatment response based on genomic signatures in blood [10], and the NETest has been introduced as a blood-based multianalyte transcript diagnostic tool for neuroendocrine tumors [11, 12]. However, blood-based RNA sequencing to investigate systemic and immune-related factors specific to [^177^Lu]Lu-DOTA-TATE response remains underexplored. Thus, this prospective study aims to identify key blood-based molecular biomarkers to predict treatment response and to uncover central molecular hub genes that may underlie variability in [^177^Lu]Lu-DOTA-TATE response.

## Materials and methods

### Subject and study design

We prospectively enrolled subjects scheduled for [^177^Lu]Lu-DOTA-TATE therapy due to metastatic neuroendocrine tumors expressing somatostatin receptors from August 2021 to July 2023. Patients had no prior PRRT and were expected to complete all four cycles of [^177^Lu]Lu-DOTA-TATE. Eligibility criteria for [^177^Lu]Lu-DOTA-TATE were based on established guidelines and the Lutathera package insert [13]. Treatment response was assessed according to RECIST 1.1 after completing all four cycles. To collect RNA samples, 3 mL of whole blood was drawn from each subject before the first cycle of [^177^Lu]Lu-DOTA-TATE. Blood samples were stored in PAXgene® Blood RNA tubes (BD Biosciences, San Jose, CA, USA). Following sample collection, we isolated buffy coats by centrifuging each sample at 800 g for 10 min. The buffy coats were then carefully transferred to labeled cryotubes using a pipette and stored at − 50 °C to preserve sample integrity. No specific enrichment step was performed to isolate circulating tumor cells.

### Baseline clinical and laboratory characteristics

Baseline demographic, clinical, and laboratory data were collected to explore potential associations with treatment response beyond transcriptomic signatures. These included patient age, sex, pre-treatment complete blood count, tumor grade, Ki-67 index, tumor origin, and prior treatment history. Prior treatments were categorized into systemic therapies (e.g., lanreotide, everolimus, sunitinib, etoposide/cisplatin) and local therapies such as radiotherapy. Continuous variables were compared using the Kruskal–Wallis test, while categorical variables were analyzed with Fisher’s exact test. Due to missing data on Ki-67 and tumor grade in the pathologic reports, three patients with paraganglioma or pheochromocytoma—one from each treatment group—were excluded from this analysis.

### RNA sequencing

Total RNA was extracted from the labeled cryotubes following the manufacturer's protocol, and RNA sequencing was subsequently performed on the buffy coats. RNA quality and concentration were measured using an Agilent 2100 Bioanalyzer (Agilent Technologies, Santa Clara, CA, USA) to ensure samples met the quality criteria for sequencing. Library preparation was conducted using the TruSeq Stranded mRNA Library Prep Kit (Illumina, San Diego, CA, USA) to selectively enrich mRNA and maintain strand specificity. Both the quality and concentration of the prepared libraries were verified on the Agilent 2100 Bioanalyzer and quantified using the Qubit 4.0 Fluorometer (Thermo Fisher Scientific, Waltham, MA, USA). Libraries were then pooled in equimolar concentrations. Sequencing was performed on the Illumina NovaSeq 6000 platform (Illumina, San Diego, CA, USA), generating 150 bp paired-end reads with a minimum sequencing depth of 30 million reads per sample to achieve comprehensive transcriptome coverage.

### RNA-seq data analysis and statistics

All the process of analysis were performed in R studio (Version 4.3.3; The R Foundation, Vienna, Austria). Although all samples were sequenced at the same facility using identical parameters and methods, they were collected over a two-year period and processed in two separate sequencing batches. This may have introduced minor processing variations. To account for potential batch effects, the Combat-seq package was applied as a precautionary measure [14]. Responders were defined as patients who showed partial response or stable disease according to RECIST1.1 criteria. Non-responders were defined as patients who showed progressive disease. Differential expression analysis was performed using DESeq2 (version 1.40.2; Bioconductor, USA). The differentially expressed genes between responders and non-responders were screened by the criteria of log fold change (logFC) ≥ 0.5 and an adjusted p-value < 0.05. Adjusted p-values were calculated using the Benjamini–Hochberg method to control for false discovery rate. To explore the biological functions and pathways associated with DEGs, we conducted enrichment analyses using Gene Ontology (GO), Kyoto Encyclopedia of Genes and Genomes (KEGG), and Reactome pathway databases. Also, Weighted Gene Co-expression Network Analysis (WGCNA) was employed to identify gene modules associated with treatment responses [15]. To achieve a suitable scale-free network, the correlation coefficient threshold was set to > 0.85. The soft-threshold power 18 is adopted to get appropriate adjacency. And we merged modules that have close expression values at a height of 0.25. All generated modules were checked to confirm correlation with treatment response and statistical threshold was an adjusted p-value < 0.05.

### Pretreatment image analysis

Administration doses for pretreatment somatostatin target PET were 185 MBq of [^68^Ga]Ga-DOTA-TOC. [^68^Ga]Ga-DOTA-TOC PETs from 25 subjects were analyzed through MIM encore (version: 7.1.7. MIM software, Cleveland, OH, USA). One subject was excluded from the PET image analysis due to the unavailability of raw image data from a PET scan that had been conducted at another clinic, which prevented access to necessary PET parameters. Considering reproducibility of imaging parameters, standardized uptake value maximum (SUVmax), standardized uptake value mean (SUVmean), and tumor volume were collected. Tumor volume was delineated by applying a threshold of 1.5 times the SUVmean of normal liver tissue [16]. And physiologic uptake was removed manually. These parameters were compared according to different treatment responses using Mann Whitney test and Kruskall Wallis test.

## Result

### Characteristic of subjects

A total of 31 patients who underwent [^177^Lu]Lu-DOTA-TATE therapy were screened. Five patients were excluded as they did not complete the four cycles of PRRT (four were lost to follow-up, and one due to financial constraints). Thus, the study ultimately included 26 participants, ranging in age from 26 to 78 years (mean age 55.5 ± 12.7 years). The cohort comprised 17 males and 9 females. Disease origins varied, with 14 cases of gastroenteropancreatic origin, 7 cases of paraganglioma/pheochromocytoma, 2 cases of thymic origin, 1 case of hepatic origin, and 2 cases of unknown origin. Based on RECIST 1.1 criteria, 6 subjects demonstrated progressive disease (PD), 12 had stable disease (SD), 8 showed a partial response (PR), and none achieved a complete response (CR). Among baseline clinical and demographic characteristics, only age demonstrated a statistically significant difference among the three response groups. The mean age was lowest in the PD group (50.8 ± 9.1 years) and highest in the PR group (63.8 ± 16.3 years) (P = 0.03). In multinomial logistic regression analysis, age corresponded to higher odds of being in the PR (OR 1.12, P = 0.061), or SD (OR 1.01, P = 0.790) groups compared to PD group. Although no other variables reached statistical significance, the Ki-67 index tended to be higher in the PD group (25.5%) compared to other groups. No significant differences were observed in white blood cell count, neutrophil or lymphocyte proportions. Tumor origin varied across patients without a distinct distribution pattern according to treatment response. Regarding prior treatments, patients received up to three different systemic therapies, including lanreotide, everolimus, sunitinib, irinotecan and etoposide/cisplatin. Local radiotherapy was performed in a total of six patients, five of whom had Paraganglioma/Pheochromocytoma. Additionally, among the seven patients with Paraganglioma/Pheochromocytoma, two had previously undergone [^131^I]I-metaiodobenzylguanidine therapy. Four patients were treatment-naive, with three in the SD group and one in the PR group. Key characteristics are summarized in Table 1.

**Table 1:** Characteristics of subjects

	Total (n = 26)	PR (n = 8)	SD (n = 12)	PD (n = 6)	P value
Age	55.5 ± 12.7	63.8 ± 16.3	52.4 ± 9.3	50.8 ± 9.1	0.030
Sex	Male: 17 Female: 9	Male: 5 Female: 3	Male: 10 Female: 2	Male: 2 Female: 4	0.140
Hemoglobin (g/dL)	12.7 ± 2.0	12.7 ± 2.3	13.0 ± 2.0	12.3 ± 2.1	0.765
WBC (× 10^6^/μL)	6.71 ± 2.08	7.18 ± 2.91	6.79 ± 1.46	5.94 ± 2.01	0.886
Neutrophil (%)	63.6 ± 11.1	65.9 ± 8.7	61.1 ± 10.3	65.2 ± 15.7	0.526
Lymphocyte (%)	25.8 ± 10.3	23.3 ± 9.3	28.6 ± 10.1	24.2 ± 12.5	0.469
Platelet (× 10^6^/μL)	242 ± 106	220 ± 83	286 ± 122	182 ± 62	0.096
Ki-67 (%)*	16.8 ± 20.1	13.8 ± 15.8	14.8 ± 17.2	25.5 ± 31.3	0.616
Tumor grade*	Grade 1 (3)	Grade 1 (0)	Grade 1 (2)	Grade 1 (1)	0.903
	Grade 2 (16)	Grade 2 (6)	Grade 2 (7)	Grade 2 (3)	
	Grade 3 (4)	Grade 3 (1)	Grade 3 (2)	Grade 3 (1)	
Origin	GEP 14	Pancreas 2	Pancreas 5	Pancreas 2	0.301
	Para/Pheo 7	Stomach 1	Rectum 2	Para/Pheo 3	
	Thymus 2	Duodenum 1	Para/Pheo 3	Unknown 1	
	Liver 1	Rectum 1	Thymus 2		
	Unknown 2	Para/Pheo 1			
		Liver 1			
		Unknown 1			
Local radiotherapy (n)	6	1	2	3	0.291
Number of systemic therapy (n)	0 (4)	0 (1)	0 (3)	0 (0)	0.845
	1 (9)	1 (3)	1 (4)	1 (2)	
	≥ 2 (13)	≥ 2 (4)	≥ 2 (5)	≥ 2 (4)	

*Ki-67 and tumor grade data were unavailable in three subjects (one for each treatment response group) with paraganglioma/pheochromocytoma (Para/Pheo)

### Imaging parameters

One patient, who belonged to the PD group, was excluded from the PET parameter analysis due to missing raw image data. The remaining 25 subjects showed relatively high somatostatin receptor expression of primary and metastatic lesions. Medians of all subjects’ SUVmax and SUVmean were 25.9 and 13.5 respectively. In the progressed disease group, SUVmax and SUVmean showed slightly lower values, but there was no statistical significance. Comparison result with treatment responses are summarized in Table 2.

**Table 2:** PET parameters and Treatment response

Parameter	Response	Subjects (n)	Median	Range	p-value
SUVmax	Partial response	8	26.4	12.8 to 75.1	0.430
	Stable disease	12	34.8	12.5 to 99.2	
	Progressive disease*	5	20.2	9 to 73.4	
SUVmean	Partial response	8	14.5	7.4 to 30.0	0.576
	Stable disease	12	14.0	6.3 to 88.6	
	Progressive disease*	5	9.9	5.9 to 18.7	
Tumor volume	Partial response	8	71.8	15.4 to 743.0	0.811
	Stable disease	12	157.0	13.6 to 385.0	
	Progressive disease*	5	188.0	4.9 to 418.0	

*One subject was excluded from PET parameter analysis owing to the unavailability of raw PET data

### Differentially expressed genes associated with response highlight IFN-signaling pathways in nonresponders

**Fig. 1:**
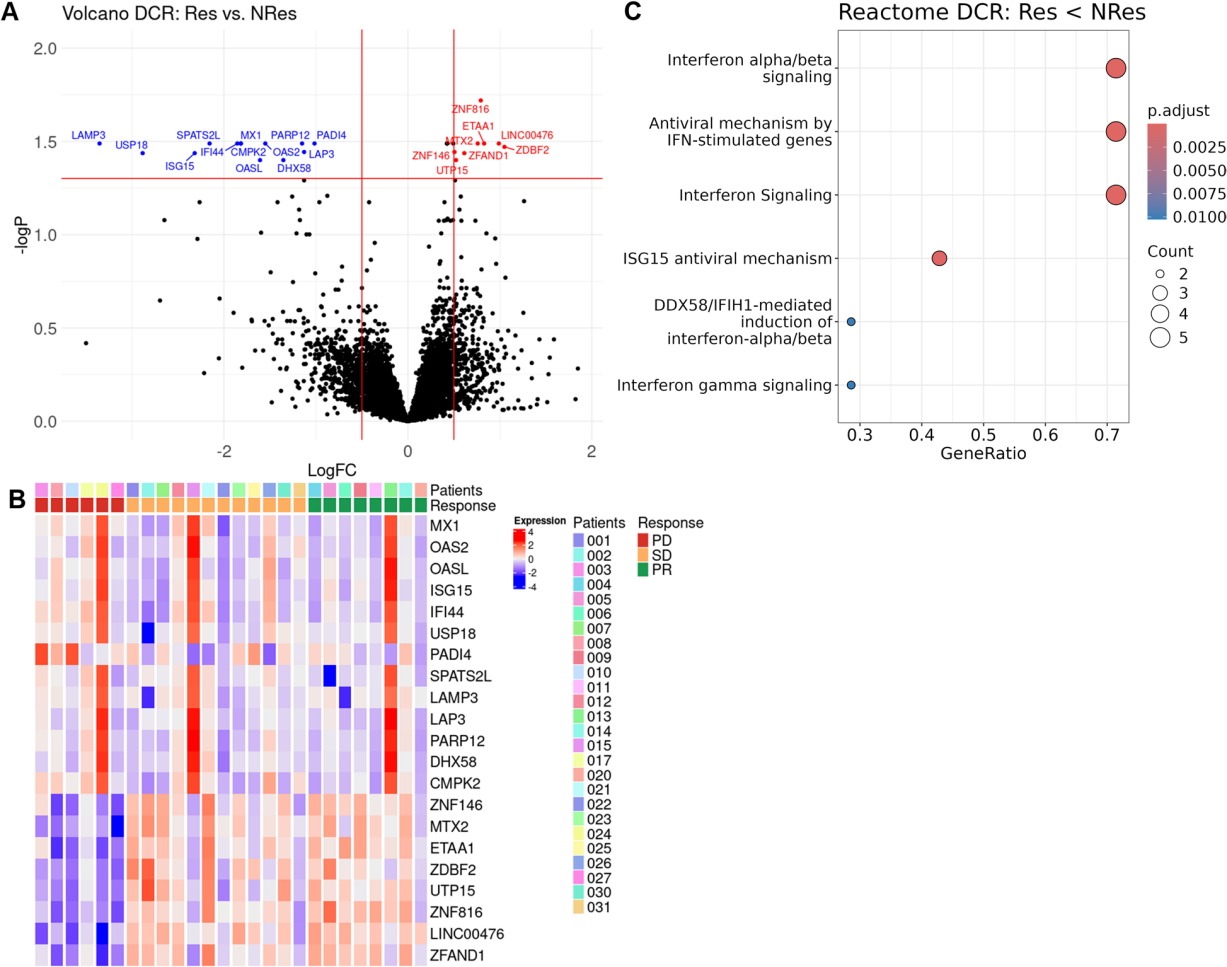
Differential gene expression and pathway analysis in treatment response groups. **A** Volcano plot showing differentially expressed genes (DEGs) in responders (Res) versus nonresponders (NRes). The x-axis represents the log fold change (LogFC) of gene expression in responders relative to nonresponders. The y-axis shows the statistical significance at the log scale. The vertical and horizontal red lines indicate the LogFC and significance thresholds used to define DEGs. **B** Heatmap of the 21 differentially expressed genes. Expression values are scaled across the patient samples, with red indicating relatively high expression and blue indicating relatively low expression. Patient IDs and response (PD, SD, or PR) are annotated above the plot. **C** Dot plot of the six most enriched Reactome pathways associated with the 13 genes that are overexpressed in nonresponders. The x-axis represents the gene ratio (the number of overlapping DEGs divided by the total number of genes in each pathway). Dot size indicates the number of overlapping genes, and color intensity shows the adjusted p-value

Batch effects were adjusted using Combat-seq, and subsequent principal component analysis (PCA) plots confirmed successful batch effect correction with clustering driven predominantly by biological variables rather than batch origin (Supplement Fig. 1). DEGs between responders and non-responders were identified (Fig. 1). The volcano plot illustrates the DEGs between responders and non-responders. The plot includes 16,051 genes, with significantly differentially expressed genes highlighted (Fig. 1a). The heatmap of 21 DEGs are in (Fig. 1b). The 13 upregulated genes at non-responders are related to “Interferon alpha/beta signaling” and “IFN signaling genes” according to the Reactome analysis (Fig. 1c). Meanwhile, no significantly enriched terms or pathways were identified with DEGs upregulated in responders.

### Weighted gene co-expressing network analysis

**Fig. 2:**
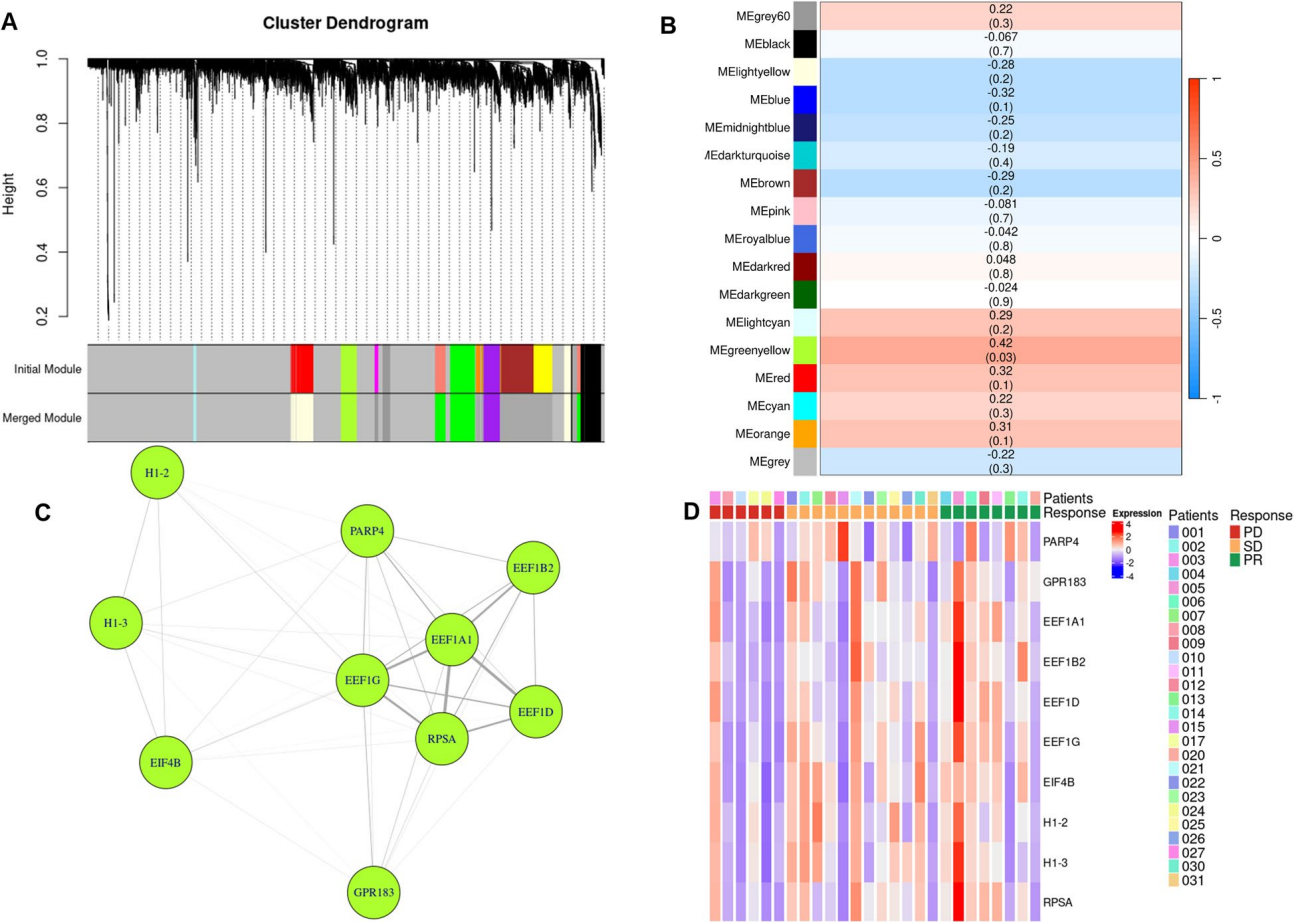
Weighted gene co-expression network analysis (WGCNA) identifies a key module associated with treatment response. **A** Gene clustering dendrogram showing assignment of modules. Hierarchical clustering of all detected genes yields the initial modules (upper color bar) and the merged modules (lower color bar). Each color represents a distinct gene co-expression modules. **B** Heatmap of Pearson correlations between each module eigengene and treatment response status. Values within each cell indicate the correlation coefficient (top) and corresponding p-value (bottom), with red denoting positive associations and blue negative associations with the response **C** Network diagram of the top 10 hub genes from the response-associated gene module, ranked by intramodular connectivity. The line thickness represents the centrality of the corresponding nodes. **D** Heatmap of 10 hub genes in the module. Expression values are scaled across the patient samples, with red indicating relatively high expression and blue indicating relatively low expression. Patient IDs and response (PD, SD, or PR) are annotated above the plot

**Fig. 3:**
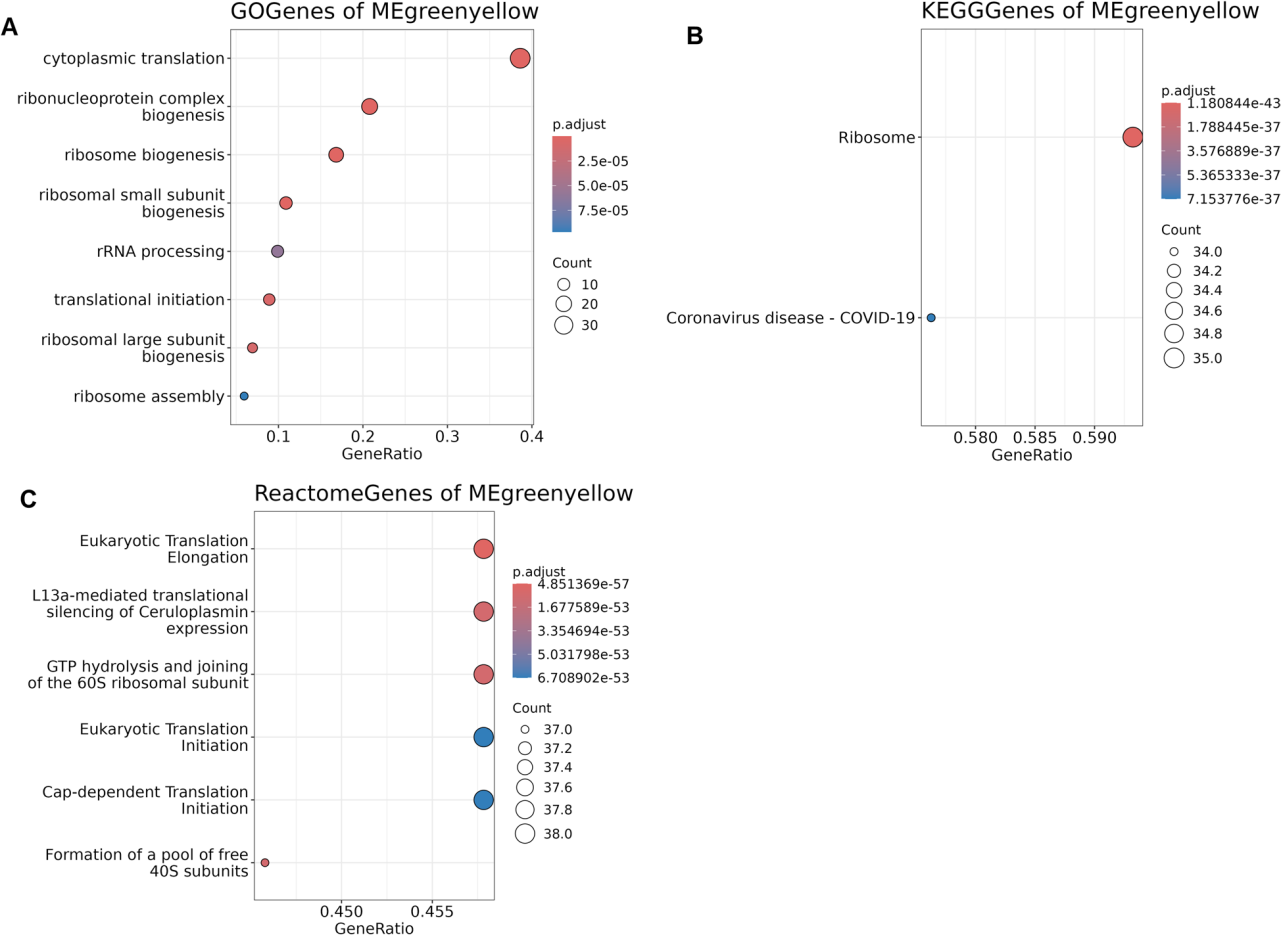
Functional enrichment of hub genes in the greenyellow module. **A** Gene Ontology, **B** KEGG analysis, **C** Reactome pathway analysis of genes in the “greenyellow” module. The top six most significant terms were visualized in the plot. The x-axis represents the gene ratio (the number of overlapping hub genes divided by the total number of genes in each pathway). Dot size indicates the number of overlapping genes, and color intensity shows the adjusted p-value. *KEGG* Kyoto Encyclopedia of Genes and Genomes

WGCNA was performed to identify key gene networks associated with response to [^177^Lu]Lu-DOTA-TATE treatment. WGCNA is a systems biology approach used to describe the correlation patterns among genes across multiple samples. By constructing networks based on gene co-expression, WGCNA allows for the identification of modules, or clusters, of highly correlated genes. WGCNA facilitated the detection of specific gene networks potentially linked to treatment response, providing insights into the molecular mechanisms underlying patients'varied responses to [^177^Lu]Lu-DOTA-TATE. The genes were grouped into 25 modules which have size from 30 to 552 genes per models. Finally 17 merged modules were obtained. We found that the only module *greenyellow* showed the significance across the treatment response. The correlation coefficient and p value of the module *greenyellow* are 0.42 and 0.03 respectively (Fig. 2a, b). Top 10 hub genes in the module *greenyellow* were identified based on topological overalap matrix and degree centrality. Their adjacency and heatmap of expression level are visualized in (Fig. 2c, d). The line thickness represents the centrality of the corresponding nodes. The expresison levels of top 10 hub genes are presented as log scale value. eEF1A1 gene presented the highest centrality. Functional enrichment analysis of the gene in module greenyellow showed that enriched GO biolobical process terms were “Cytoplasmic translation” and “Ribosome biogenesis”. Also in the KEGG, and reactome analysis, the genes in module greenyellow related to “Ribosome” and “Protein translation” respectively (Fig. 3).

## Dicussion

In this prospective study, we demonstrated that systemic molecular factors, as determined by blood transcriptome levels, are associated with the response to [^177^Lu]Lu-DOTA-TATE. We identified blood molecular biomarkers associated with therapeutic response through RNA sequencing of metastatic NET patients prior to four cycles of [^177^Lu]Lu-DOTA-TATE therapy. Notably, since all patients were eligible for [^177^Lu]Lu-DOTA-TATE treatment, demonstrating high uptake in pretreatment PET, there were no significant differences in pretreatment PET parameters among treatment responses. Similarly, there were some reports that SUV showed no definite advantage for prediction of treatment response [17, 18]. Pretreatment PET can evaluate SSTR2 expression and assess eligibility of [^177^Lu]Lu-DOTA-TATE therapy. However, PET parameters like SUV or tumor volume that calculated in different methods seem like they have little role as a biomarker to predict treatment responses. When comparing various baseline clinical and demographic characteristics, their role as predictive biomarkers also seems modest. It is important to note, however, that our study is limited by a relatively small sample size and the reliance on a single baseline assessment, which may not fully capture the dynamic nature of tumor biology and host factors. Nonetheless, the observation that the PD group exhibited a higher Ki-67 index alongside slightly lower SUVmax and SUVmean highlights the complexity of treatment response prediction. These results suggest that a comprehensive evaluation incorporating multiple clinical, pathological, and imaging parameters is warranted to better stratify patients and optimize therapeutic decision-making. Overexpressed genes in non-responders were linked to the innate immune system. Our findings suggest a potential role for systemic immune reactions in influencing [^177^Lu]Lu-DOTA-TATE therapy outcomes in NET, even among patients with high SSTR expression consistent with current indications.

Responders were defined as patients who showed partial response or stable disease, while non-responders were defined as those who exhibited progressive disease. [^177^Lu]Lu-DOTA-TATE therapy was aimed at patients with metastatic disease who had progressive disease despite prior treatment. Therefore, stable disease is considered a valuable outcome, as it demonstrates therapeutic benefit by stabilizing the disease. However, a more stringent approach may be necessary in future studies.

Differentially expressed genes in non-responders to [^177^Lu]Lu-DOTA-TATE therapy were notably associated with interferon-gamma (INF-γ) signaling, suggesting that the innate immune response plays a crucial role in mediating resistance to therapy. IFN-γ is a key cytokine in the immune system, primarily produced by activated T cells and NK cells, and it is pivotal in modulating tumor-immune interactions. It orchestrates anti-tumor immunity by enhancing antigen presentation through upregulation of major histocompatibility complex (MHC) molecules and promoting the recruitment and activation of effector immune cells. However, paradoxically, IFN-γ can also contribute to immune evasion and resistance mechanisms in the tumor microenvironment [19]. One possible mechanism by which IFN-γ signaling may contribute to resistance is through its ability to induce the expression of immune checkpoint molecules such as PD-L1 on tumor cells, creating an immunosuppressive microenvironment that dampens the cytotoxic activity of immune cells [20, 21]. Additionally, sustained IFN-γ signaling can promote tumor cell adaptation by triggering the expression of genes involved in survival pathways and resistance to apoptosis [22]. In the context of [^177^Lu]Lu-DOTA-TATE therapy, IFN-γ signaling may influence therapy resistance through several mechanisms. One feasible mechanism is that chronic immune activation driven by IFN-γ could alter the tumor microenvironment, leading to the recruitment of pro-tumorigenic immune cells or suppression of effective anti-tumor immunity. As another mechanism of crosstalk, the association of IFN-γ signaling with innate immune responses suggests that non-responders may have a tumor microenvironment characterized by heightened inflammation, which could interfere with the cytotoxic effects of PRRT. In this aspect, [^177^Lu]Lu-DOTA-TATE not only exerts its therapeutic effect through radiation-mediated cell killing but also plays a crucial role in modulating tumor immunology, which significantly impacts treatment response.

Gene network analysis revealed that hub genes associated with treatment response were significantly enriched in a module linked to cytoplasmic translation processes and ribonucleoprotein complex biogenesis. eEF1A1 is a key component of the translational machinery, responsible for delivering aminoacyl-tRNA to the ribosome during protein synthesis [23]. eEF1A1 coordinates heat shock proteins so that it could relate to regulation of processes that control essential cell function under stress [24]. Other studies reported ribosome biogenesis, including eEF1A1, may play an important role in tumor, but its expression varies in different tumors [25, 26]. Ribosome biogenesis has been linked to cancer cell proliferation, with hypertrophic nucleoli and increased ribosomal output commonly observed in aggressive tumors [27]. In our context, the upregulation of translation-related processes appears to correlate with enhanced sensitivity to therapy rather than resistance particularly in blood samples. This might be due to the dependency of [^177^Lu]Lu-DOTA-TATE efficacy on somatostatin receptor expression, which may be better sustained in metabolically active cells with robust protein synthesis machinery. The findings also raise intriguing hypotheses regarding the role of ribosomal biogenesis and cytoplasmic translation in modulating tumor-immune interactions. Enhanced translation activity could increase the production of immunomodulatory proteins, potentially amplifying immune-mediated cytotoxicity in the tumor microenvironment [28, 29]. This aligns with evidence suggesting that active ribosomal function is not merely a hallmark of proliferation but also a facilitator of the dynamic interplay between tumors and the immune system, considering the results of IFN-gamma signaling associated with the response.

Another component, PARP4 genes, also known as vault poly(ADP-ribose) polymerase (vPARP) is one of the hub gene of the same module. PARP4 gene is subfamily of PARP genes and its key functional motifs is BRCA1 C terminus [30, 31]. So the function of PARP4 is little known but we can suspect it is involved in the DNA repair pathway. Additionally, there are some results that PARP4 might have a tumor-suppressor function [32]. Similarly, we also reveal the module have PARP4 as a hub gene shows high level of expression in patients who showed good treatment response to [^177^Lu]Lu-DOTA-TATE therapy. Considering our results are obtained from subjects who went through [^177^Lu]Lu-DOTA-TATE therapy, these findings can let us understand that how we got better treatment response from subjects who showed higher expression level of the module than those who did not.

This study has some limitations. First, the sample size was relatively small, which may limit the generalizability of our findings and necessitates validation in larger, independent cohorts. Second, while blood transcriptomic profiling provides valuable systemic insights, it does not capture the spatial and microenvironmental heterogeneity of tumor-intrinsic factors, which may also influence treatment response. Third, despite the identification of IFN-signaling pathways in non-responders, functional validation studies are needed to confirm the mechanistic role of these pathways in resistance to [^177^Lu]Lu-DOTA-TATE therapy. Future studies integrating tumor tissue analysis, serial blood sampling, and functional assays will be essential to refine these biomarkers and better understand their clinical implications.

The findings of this study highlight the significant role of systemic effects and inflammatory responses in shaping the therapeutic outcomes of [^177^Lu]Lu-DOTA-TATE treatment for neuroendocrine tumors. These results support the potential of blood-based transcriptomic biomarkers as predictive tools for assessing treatment response. Furthermore, the study suggests that integrating systemic therapies, such as immuno-oncology treatments, could address limitations of [^177^Lu]Lu-DOTA-TATE monotherapy and enhance overall efficacy. While the identified predictive modules and other findings require further validation in diverse cohorts, they represent promising insights into developing more personalized and effective therapeutic strategies.

## Conclusions

Our findings suggest that peripheral blood transcriptomic profiles reflect immune-related mechanisms associated with response to [^177^Lu]Lu-DOTA-TATE therapy. The identification of a hub gene linked to treatment outcome reinforces the potential of blood-based biomarkers in predicting therapeutic efficacy. Although further validation in larger, prospective cohorts is warranted, these results lay the groundwork for integrating immune transcriptomic markers into clinical decision-making and tailoring treatment strategies for patients with neuroendocrine tumors.

## Supplementary Information

Supplementary Material 1

## Data Availability

The datasets generated during and/or analysed during the current study are available from the corresponding author on reasonable request.

## Abbreviations

NETs: Neuroendocrine tumors
DEGs: Differentially expressed genes
WGCNA: Weighted Gene Co-expression Network Analysis
PRRT: Peptide receptor radionuclide therapy
SSTR: Somatostatin receptors
GEP: Gastroenteropancreatic
PET: Positron emission tomography
GO: Gene Ontology
KEGG: Kyoto Encyclopedia of Genes and Genomes
SUVmax: Standardized uptake value maximum
SUVmaen: Standardized uptake value mean
PD: Progressive disease
SD: Stable disease
PR: Partial response
CR: Complete response
INF-γ: Interferon-gamma
MHC: Major histocompatibility complex
vPARP: Vault poly(ADP-ribose) polymerase
