# Supplementary material for: Blood-based transcriptomic biomarkers for response to [177Lu]Lu-DOTA-TATE therapy in neuroendocrine tumors
Source: EJNMMI Res. 2025 Aug 2;15:100. doi: 10.1186/s13550-025-01284-w (PMC12317932; doi:10.1186/s13550-025-01284-w)
Supplement: Supplementary file 1 — Supplementary Material 1 [file 13550_2025_1284_MOESM1_ESM.docx]

**Blood-Based Transcriptomic Biomarkers for Response to [^177^Lu]Lu-DOTATATE Therapy in Neuroendocrine Tumors;** Supplement data


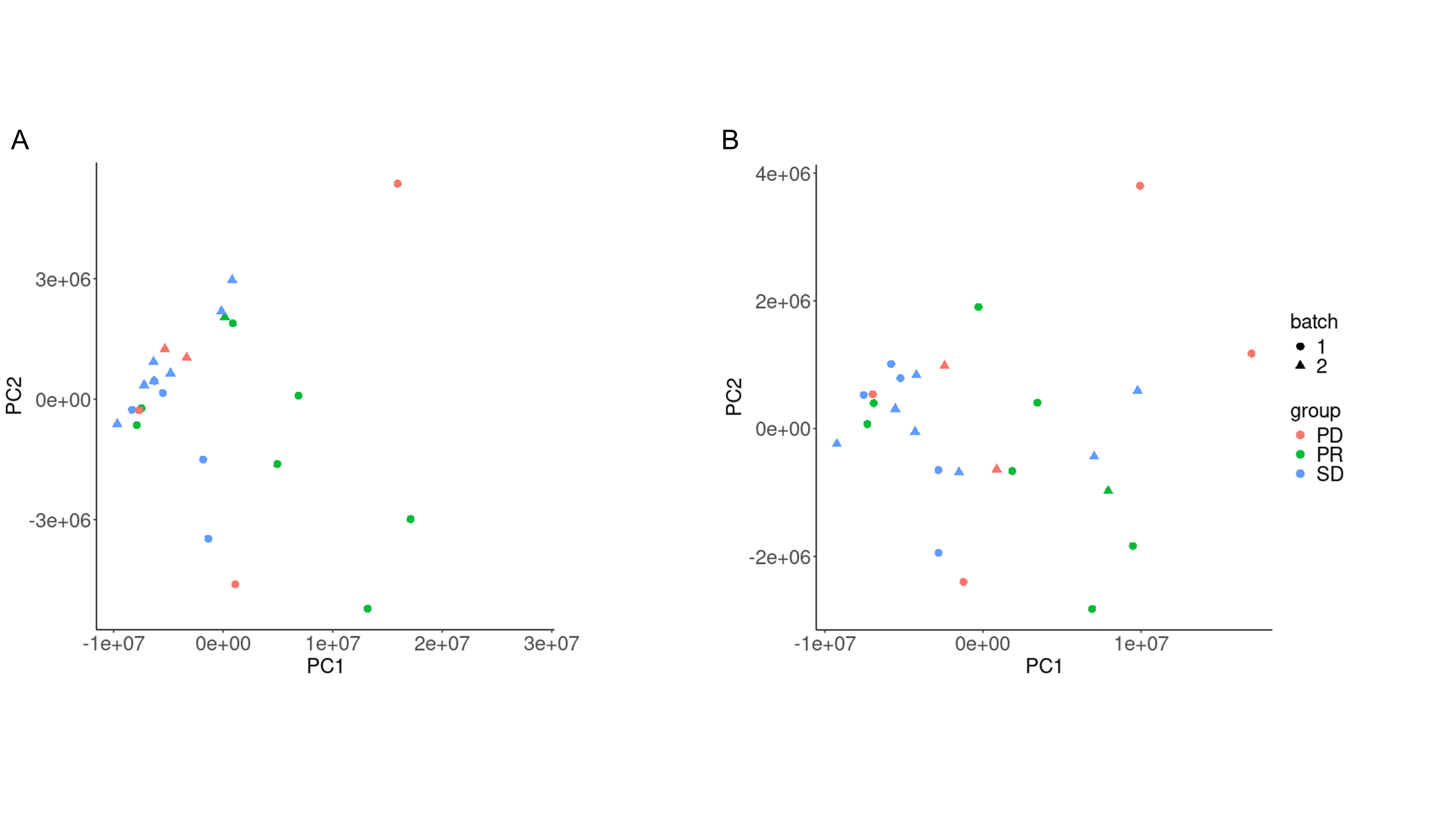


Supplement Figure 1. Principlal component analysis, (A) Before and (B) after batch correction.
